# A genome-wide study of two-component signal transduction systems in eight newly sequenced mutans streptococci strains

**DOI:** 10.1186/1471-2164-13-128

**Published:** 2012-04-04

**Authors:** Lifu Song, Padhmanand Sudhakar, Wei Wang, Georg Conrads, Anke Brock, Jibin Sun, Irene Wagner-Döbler, An-Ping Zeng

**Affiliations:** 1 Institute of Bioprocess and Biosystems Engineering, Hamburg University of Technology, Hamburg, Germany; 2 Division of Oral Microbiology and Immunology, Department of Operative and Preventive Dentistry & Periodontology, RWTH Aachen University, Aachen, Germany; 3 Group Microbial Communication, Division of Microbial Pathogenesis, Helmholtz-Centre for Infection Research, Inhoffenstr 7, 38124, Braunschweig, Germany; 4 Tianjin Institute of Industrial Biotechnology, Chinese Academy of Science, Tianjin 300308, Peoples Republic of China

**Keywords:** Mutans streptococci, *Streptococcus mutans*, *Streptococcus ratti*, *Streptococcus sobrinus*, Two-component system, Histidine kinase, Response regulator, Signal transduction, Comparative genomics

## Abstract

**Background:**

Mutans streptococci are a group of gram-positive bacteria including the primary cariogenic dental pathogen *Streptococcus mutans *and closely related species. Two component systems (TCSs) composed of a signal sensing histidine kinase (HK) and a response regulator (RR) play key roles in pathogenicity, but have not been comparatively studied for these oral bacterial pathogens.

**Results:**

HKs and RRs of 8 newly sequenced mutans streptococci strains, including *S. sobrinus *DSM20742, *S. ratti *DSM20564 and six *S. mutans *strains, were identified and compared to the TCSs of *S. mutans *UA159 and NN2025, two previously genome sequenced *S. mutans *strains. Ortholog analysis revealed 18 TCS clusters (HK-RR pairs), 2 orphan HKs and 2 orphan RRs, of which 8 TCS clusters were common to all 10 strains, 6 were absent in one or more strains, and the other 4 were exclusive to individual strains. Further classification of the predicted HKs and RRs revealed interesting aspects of their putative functions. While TCS complements were comparable within the six *S. mutans *strains, *S. sobrinus *DSM20742 lacked TCSs possibly involved in acid tolerance and fructan catabolism, and *S. ratti *DSM20564 possessed 3 unique TCSs but lacked the quorum-sensing related TCS (ComDE). Selected computational predictions were verified by PCR experiments.

**Conclusions:**

Differences in the TCS repertoires of mutans streptococci strains, especially those of *S. sobrinus *and *S. ratti *in comparison to *S. mutans*, imply differences in their response mechanisms for survival in the dynamic oral environment. This genomic level study of TCSs should help in understanding the pathogenicity of these mutans streptococci strains.

## Background

A group of oral streptococci which is closely related to the primary cariogenic dental pathogen *Streptococcus mutans *is referred to as "mutans streptococci", and it includes in addition to *S. mutans *also *S. ratti*, and *S. sobrinus *among others. A common feature of the mutans streptococci is their ability to metabolize carbohydrates present in our daily diet to produce energy for their survival and to produce organic acids which erode and dematerialize the tooth enamel and dentin. These oral bacteria are able to grow in a community and colonize the oral environment by attaching to the tooth surfaces and forming biofilms. They can also tolerate and adapt to the harsh and rapidly changing physiological conditions of the oral cavity such as extreme acidity, fluctuation of nutrients, reactive oxygen species, and other environmental stresses [1]. They occasionally also cause bacteremia and infective endocarditis [2]. All together, the survival and adaptation of mutans streptococci is directly linked to their virulence and pathogenicity.

Bacterial two-component signal transduction systems (TCS) play important roles for many bacteria by enabling them to detect and respond to diverse changes/stresses in the environment. The conspicuous absence of TCS proteins in mammalian genomes makes them interesting potential targets for the development of novel anti-bacterial drugs. A bacterial two-component system comprises in general a transmembrane sensor histidine kinase (HK) and a corresponding cytoplasmic response regulator (RR) encoded by genes located adjacently within the same operon, although stand-alone genes coding for HKs or RRs (without a corresponding cognate HK/RR in the same operon) have also been reported. In some cases, a HK and a RR are found to be merged in the same polypeptide, giving rise to a so called 'hybrid' HK protein. A HK protein is autophosphorylated at its conserved histidine (His) residue upon the recognition of a specific environmental stimulus. The phosphoryl group is then transferred to the aspartate (Asp) residue of the corresponding response regulator [3]. While HKs in general serve to detect signals, the most common function of the RRs is to bind directly to DNA and thereby modulating the expression of a certain set of genes which are necessary for mounting a physiological response to the perceived signals [3]. HK and RR proteins are composed of domains which are structurally and functionally conserved and can be used for their classification.

With the advance of large scale sequencing technologies and bioinformatics tools, it has become possible to computationally predict the putative functions of genes/proteins from the whole genome of an organism. The prediction of TCS proteins using a whole genome-based computational approach has been carried out for different organisms. Such *in silico *studies have broadened our understanding of genomic repertoires essential for the growth and adjustment of the organisms to altering environmental challenges [4]. The genome sequencing and annotation of the first *S. mutans *strain (UA159, serotype c) has paved the way for researchers to carry out numerous molecular biological and functional genomic studies that help in understanding the robustness, genetic specificity and complexity of this bacterium as an oral pathogen [5]. 14 TCS clusters have previously been identified in *S. mutans *UA159 [5,6] and many of them have been reported to be involved in its virulence, adaptation and survival [6-10]. After the sequencing of six *S. mutans *isolates, namely *S. mutans *5 DC8, KK21, KK23, AC4446, ATCC25175 and NCTC11060, as well as *S. ratti *DSM20564 and *S. sobrinus *DSM20742, we performed a systematic identification and classification of putative TCS proteins in the genomes of these eight mutans streptococci strains, using the well annotated genome of *S. mutans *UA159, as well as that of *S. mutans *NN2025, as references. Furthermore, we conducted a thorough comparative analysis of the identified TCS proteins among these strains which provides valuable insights into the conservation and divergence of TCS proteins in the mutans streptococci strains studied here. PCR experiments have been carried out to verify the presence of several predicted TCS genes of particular interest.

## Results and discussion

### Identification of TCS proteins of the mutans streptococci strains

We have sequenced the genomes of eight mutans streptococci strains (Table 1) using the Solexa sequencing technology (genome information available at: http://134.28.64.65/www/index.php/genome/). The "high-quality draft" genomes obtained according to the definition by Chain et al. [11] have an overall genome coverage of > 99%, taking *S. mutans *NN2025 and UA159 as references. By combining the Hidden Markov Model (HMM) profiling results and the information on putative operon organization, repertoires of potential TCS proteins (HKs and RRs) in the eight newly sequenced mutans streptococci strains were obtained, as shown in Table 2 in comparison to *S. mutans *NN2025 and UA159. The total numbers of TCS proteins identified are comparable among the 10 mutans streptococci strains.

**Table 1:** Eight newly sequenced mutans streptococci strains

Species	Strain	Short description
*S. mutans*	5 DC8	Serotype c, isolated from root caries by David Beighton (London, UK), alterations in 16S sequence in comparison to type strain.

*S. mutans*	AC4446	Serotype c, isolated from a proven case of infective endocarditis in Dillingen (Germany).

*S. mutans*	KK21	Serotype c, isolated from enamel caries of an adult by Susanne Kneist (Jena, Germany), potent producer of bacteriocin.

*S. mutans*	KK23	Serotype c, isolated from enamel caries of a child by Susanne Kneist (Jena, Germany), potent producer of bacteriocin.

*S. mutans*	ATCC25175	Type strain, serotype c, isolated from carious dentine, quality control strain.

*S. mutans*	NCTC11060	Serotype f, isolated in Denmark from a patient's blood (bacteremia), reference strain.

*S. ratti*	DSM20564	Type strain (= ATCC19645), serotype b, isolated from caries lesion in rat, nearest neighbor to species *S. mutans *with a 94-95% similarity on 16S level.

*S. sobrinus*	DSM20742	Type strain (= ATCC33478), serotype non-d & non-g, isolated from human dental plaque, 93% similarity with *S. mutans *on 16S level; considered as a relevant cariogenic species in human.

The species identities were determined by appliying biochemical testing (ATB 32 Strep) together with complete 16S rRNA gene sequencing.

**Table 2:** Identification and classification of putative two component systems in the eight newly sequenced mutans streptococci strains

Strain	S. mutans UA159	S. mutans NN2025	S. mutans 5 DC8	S. mutans KK21	S. mutans KK23	S. mutans AC4446	*S. mutans *ATCC25175	*S. mutans *NCTC11060	*S. ratti* DSM20564	*S. sobrinus* DSM20742
**Identification**										
**Total TCS proteins**	29	29	29	29	29	27	25	27	28	21
**Total paired HKs**	14	14	14	14	14	13	12	13	13	9
**Orphan HKs**	0	0	0	0	0	0	0	0	1	1
**Total paired RRs**	14	14	14	14	14	13	12	13	13	9
**Orphan RRs**	1	1	1	1	1	1	1	1	1	2
**Classification**										
**HK type**										
**HPK1**	8	8	8	8	8	8	7	7	9	5
**HPK7**	3	3	3	3	3	3	3	4	3	2
**HPK8**	1	1	1	1	1	1	1	1	1	1
**HPK10**	1	1	1	1	1	1	1	1	0	1
**HPK11**	0	0	0	0	0	0	0	0	1	0
**unclassified**	1	1	1	1	1	0	0	0	0	1
**RR type**										
**NarL**	3	3	3	3	3	3	3	4	3	2
**LytTR**	2	2	2	2	2	2	2	2	1	3
**AmiR**	0	0	0	0	0	0	0	0	1	0
**OmpR**	9	9	9	9	9	9	8	8	9	6
**unclassified**	1	1	1	1	1	0	0	0	0	0

Results are presented in comparison to *S. mutans *UA159 and NN2025 as two reference strains. HKs and RRs are classified based on homology box analysis and output domain architecture, respectively. HPK1, HPK7, HPK8, HPK10, HPK11 represent HK families as found in the classification by Grebe et al. [12].

**Figure 1:**
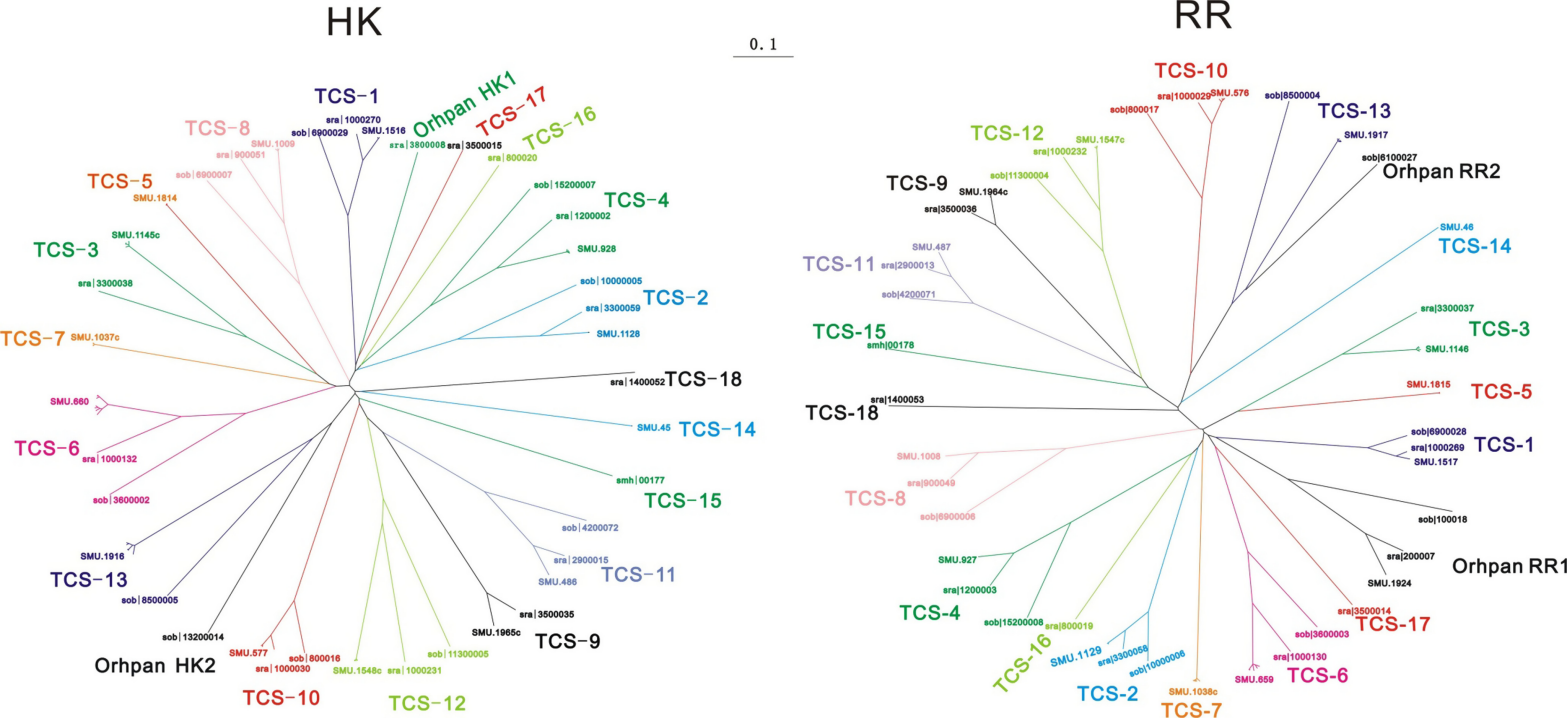
**Phylogenetic trees of the identified putative HKs and RRs in the eight newly sequenced mutans streptococci strains and the two reference strains *S. mutans *UA159 and NN2025**. The trees were constructed using ClustalX by applying the neighbor-joining method. The scale bar is shown above the trees and the scale is in units of "substitution/site".

By analyzing the putative operon organizations of genes encoding the identified TCS proteins 98.5% of the total putative HKs and 92.2% of the total putative RR were found to constitute HK-RR pairs. Ortholog analysis of the paired or non-paired TCS proteins among the 10 mutans streptococci strains revealed a total of 18 different TCS clusters, 2 orphan HKs and 2 orphan RRs (Table 3). The numbering of the TCS clusters was based on an existing numbering system used by Levesque [13], and extended to the new TCS clusters identified in this study. Co-evolution of TCS proteins could be clearly observed. This means, HKs and RRs which belong to a particular TCS cluster are usually co-present or co-absent in a specific strain. In addition, putative alleles/orthologs of the corresponding HKs and RRs were found to be highly conserved (similarity ≥ 95%) among the *S. mutans *strains. But across the species the conservation was clearly lower. Furthermore, it is obvious that, in most cases, putative HK alleles/orthologs within one TCS cluster exhibited a higher degree of diversity than the corresponding putative RR alleles/orthologs across the species. This could be attributed to the high variability of sensing/input domains harbored by the individual HKs, as will be discussed later in the classification of HKs and RRs. A clear clustering of putative HK and RR alleles/orthologs can be visualized in the phylogenetic trees shown in Figure 1, which additionally illustrates the relationships between the different TCS clusters.

**Table 3:** Ortholog analysis and classifications of the putative TCS proteins

	TCSProtein	RR family	C1	C2	*S. mutans* UA159	*S. mutans* NN2025	*S. mutans* 5 DC8	*S. mutans* KK21	*S. mutans* KK23	*S.mutans* AC4446	*S. mutans* ATCC 25175	*S. mutans* NCTC 11060	*S.ratti* DSM20564	*S. sobrinus* DSM20742
**TCS-1**	**HK (VicK)**	-	HPK1	C	SMU.1516	GI|290580114	smc|01510	smd|01560	sme|01514	smf|01497	smg|01519	smh|01557	sra|1000270(*90*)	sob|6900029(*74*)
	
	**RR (VicR)**	OmpR	-		SMU.1517	GI|290580113	smc|01511	smd|01561	sme|01515	smf|01498	smg|01520	smh|01558	sra|1000269(*96*)	sob|6900028(*87*)

**TCS-2**	**HK (CiaH)**	-	HPK1	E	SMU.1128	GI|290580439	smc|01121	smd|01170	sme|01262	smf|01137	smg|01147	smh|01166	sra|3300059(*83*)	sob|10000005(*53*)
	
	**RR (CiaR)**	OmpR	-		SMU.1129	GI|290580438	smc|01122	smd|01171	sme|01263	smf|01138	smg|01148	smh|01167	sra|3300058(*94*)	sob|10000006(*86*)

**TCS-3**	**HK (CovS)**	-	HPK1	E	SMU.1145c	GI|290580424	smc|01138	smd|01187	sme|01279	smf|01154	smg|01163	smh|01183	sra|3300038(*48*)	Absent
	
	**RR (CovR)**	OmpR	-		SMU.1146c	GI|290580423	smc|01139	smd|01188	sme|01280	smf|01155	smg|01164	smh|01184	sra|3300037(*76*)	Absent

**TCS-4**	**HK (KinF)**	-	HPK1	E	SMU.928	GI|290580625	smc|00919	smd|00964	sme|01045	smf|00943	smg|00948	smh|00938	sra|1200002(*75*)	sob|15200007(*50*)
	
	**RR (LlrF)**	OmpR	-		SMU.927	GI|290580626	smc|00918	smd|00963	sme|01044	smf|00942	smg|00947	smh|00937	sra|1200003(*89*)	sob|15200008(*70*)

**TCS-5**	**HK (ScnK)**	-	HPK1	E	SMU.1814	GI|290579846	smc|01808	smd|01870	sme|01810	smf|01679	Absent	Absent	Absent	Absent
	
	**RR (ScnR)**	OmpR	-		SMU.1815	GI|290579845	smc|01809	smd|01871	sme|01811	smf|01680	Absent	Absent	Absent	Absent

**TCS-6**	**HK (SpaK)**	-	HPK1	E	SMU.660	GI|290580857	smc|00645	smd|00684	sme|00777	smf|00640	smg|00672	smh|00668	sra|1000132(*71*)	sob|3600002(*47*)
	
	**RR (SpaR)**	OmpR	-		SMU.659	GI|290580858	smc|00643	smd|00682	sme|00775	smf|00639	smg|00670	smh|00666	sra|1000130(*80*)	sob|3600003(*67*)

**TCS-7**	**HK (PhoR)**	-	HPK1	E	SMU.1037c	GI|290580522	smc|01032	smd|01081	sme|01153	smf|01049	smg|01055	smh|01052	Absent	Absent
	
	**RR (YcbL)**	OmpR	-		SMU.1038c	GI|290580521	smc|01033	smd|01082	sme|01154	smf|01050	smg|01056	smh|01053	Absent	Absent

**TCS-8**	**HK (KinG)**	-	HPK1	M	SMU.1009	GI|290580539	smc|01005	smd|01053	sme|01135	smf|01030	smg|01036	smh|01024	sra|900051(*80*)	sob|6900007(*55*)
	
	**RR (LlrG)**	OmpR	-		SMU.1008	GI|290580540	smc|01004	smd|01052	sme|01134	smf|01029	smg|01035	smh|01023	sra|900049(*89*)	sob|6900006(*64*)

**TCS-9**	**HK (LevS)**	-	HPK7	M	SMU.1965c	GI|290579718	smc|01957	smd|02016	sme|01953	smf|01824	smg|01946	smh|01994	sra|3500035(*88*)	Absent
	
	**RR (LevR)**	NarL	-		SMU.1964c	GI|290579719	smc|01956	smd|02015	sme|01952	smf|01823	smg|01945	smh|01993	sra|3500036(*91*)	Absent

**TCS-10**	**HK (LytS)**	-	HPK8	M	SMU.577	GI|290580924	smc|00560	smd|00598	sme|00685	smf|00568	smg|00588	smh|00583	sra|1000030(*95*)	sob|800016(*80*)
	
	**RR (LytT)**	LytTR	-		SMU.576	GI|290580925	smc|00559	smd|00597	sme|00684	smf|00567	smg|00587	smh|00582	sra|1000029(*92*)	sob|800017(*70*)

**TCS-11**	**HK (LiaS)**	-	HPK7	M	SMU.486	GI|290581011	smc|00465	smd|00505	sme|00595	smf|00473	smg|00493	smh|00475	sra|2900015(*89*)	sob|4200072(*64*)
	
	**RR (LiaR)**	NarL	-		SMU.487	GI|290581010	smc|00466	smd|00506	sme|00596	smf|00474	smg|00494	smh|00476	sra|2900013(*92*)	sob|4200071(*82*)

**TCS-12**	**HK (HK11)**	-	HPK7	M	SMU.1548c	GI|290580085	smc|01542	smd|01591	sme|01547	smf|01527	smg|01550	smh|01590	sra|1000231(*53*)	sob|11300005(*50*)
	
	**RR (RR11)**	NarL	-		SMU.1547c	GI|290580086	smc|01541	smd|01590	sme|01545	smf|01526	smg|01548	smh|01588	sra|1000232(*82*)	sob|11300004(*72*)

**TCS-13**	**HK(ComD)**	-	HPK10	M	SMU.1916	GI|290579761	smc|01910	smd|01968	sme|01906	smf|01777	smg|01900	smh|01947	Absent	sob|8500005(*37*)
	
	**RR (ComE)**	LytTR	-		SMU.1917	GI|290579760	smc|01911	smd|01969	sme|01907	smf|01778	smg|01901	smh|01948	Absent	sob|8500004(*43*)

**TCS-14**	**HK**	-	?	C	SMU.45	GI|290579565	smc|00046	smd|00053	sme|00049	Absent	Absent	Absent	Absent	Absent
	
	**RR**	?	-		SMU.46	GI|290579566	smc|00047	smd|00054	sme|00050	Absent	Absent	Absent	Absent	Absent

**TCS-15**	**HK(ComP)**	-	HPK7	M	Absent	Absent	Absent	Absent	Absent	Absent	Absent	smh|00177	Absent	Absent
	
	**RR(CmpR)**	NarL	-		Absent	Absent	Absent	Absent	Absent	Absent	Absent	smh|00178	Absent	Absent

**TCS-16**	**HK**		HPK1	E	Absent	Absent	Absent	Absent	Absent	Absent	Absent	Absent	sra|800020	Absent
	
	**RR**	OmpR			Absent	Absent	Absent	Absent	Absent	Absent	Absent	Absent	sra|800019	Absent

**TCS-17**	**HK**		HPK1	E	Absent	Absent	Absent	Absent	Absent	Absent	Absent	Absent	sra|3500015	Absent
	
	**RR**	OmpR			Absent	Absent	Absent	Absent	Absent	Absent	Absent	Absent	sra|3500014	Absent

**TCS-18**	**HK**		HPK11	C	Absent	Absent	Absent	Absent	Absent	Absent	Absent	Absent	sra|1400052	Absent
	
	**RR**	AmiR			Absent	Absent	Absent	Absent	Absent	Absent	Absent	Absent	sra|1400053	Absent

**Orphan HK1**	**HK**		HPK1	N	Absent	Absent	Absent	Absent	Absent	Absent	Absent	Absent	sra|3800008	Absent

**Orphan HK2**	**HK**		?	M	Absent	Absent	Absent	Absent	Absent	Absent	Absent	Absent	Absent	sob|13200014

**Orphan RR1**	**RR (GcrR)**	OmpR	-		SMU.1924	GI|290579753	smc|01920	smd|01978	sme|01916	smf|01787	smg|01910	smh|01957	sra|200007(*87*)	sob|100018(*55*)

**Orphan RR2**		LytTR			Absent	Absent	Absent	Absent	Absent	Absent	Absent	Absent	Absent	sob|14100005

HKs are classified by two different approaches based on the homology-box analysis (classification denoted by C1) and the topology feature analysis (classification denoted by C2), respectively; RRs are classified based on the structure of their output domains (Abbreviations: *HPK*, histidine protein kinase; *C*, *E *and *M *stand for cytoplasmic, extracytoplasmic and membrane sensing, respectively; *N *denotes that no sensing mechanism could be predicted either due to the complete absence or a truncation of the predicted sensory domain; *SMU *represents the proteins corresponding to the ORFs of *S. mutans *UA159, *GI *of *S. mutans *NN2025; smc of *S. mutans *5 DC8; smd of *S. mutans *KK21; sme of *S. mutans *KK23; smf of *S. mutans *AC4446; smg of *S. mutans *ATCC 25175; smh of *S. mutans *NCTC 11060; sra of *S. ratti *DSM 20564; sob of *S. sobrinus *DSM 20742. The italic numbers in parentheses stand for similarities in percentage of the TCS proteins in *S. ratti *DSM20564 and *S. sobrinus *DSM20742 compared to the corresponding orthologs in *S. mutans *UA159. All other abbreviations are referred to in Table 2).

Eight of the 18 TCS clusters (designated as TCS-1, TCS-2, TCS-4, TCS-6, TCS-8, TCS-10, TCS-11, TCS-12) were common to all the strains. Six clusters (TCS-3, TCS-5, TCS-7, TCS-9, TCS-13, TCS-14) were observed to be absent in one or several strains. An interesting observation was that *S. ratti *DSM20564 lacked any homologs of the sensor histidine kinase and the cognate response regulator of TCS-13 (known as ComD and ComE for *S. mutans *UA159). On the other hand, TCS-16, TCS-17 and TCS-18 were found to be unique to *S. ratti *DSM20564. TCS-15, a putative ortholog of a quorum sensing-related TCS cluster well studied in the gram-positive bacterium *Bacillus subtilis*, was uniquely identified in *S. mutans *NCTC11060, a serotype f blood isolate.

Two orphan HKs were identified as uniquely present in *S. ratti *DSM20564 (sra|3800008, named as orphan HK1) and *S. sobrinus *DSM20742 (sob|13200014, orphan HK2), respectively. In contrast, an orphan RR (orphan RR1, SMU.1924 in *S. mutans *UA159) was found to be common to all the 10 strains. *S. sobrinus *DSM20742 harbored an additional unique orphan RR (sob|14100005, orphan RR2).

No hybrid HKs could be detected in all the genomes of the 10 mutans streptococci strains compared in this study. Also, the more complex variant of the two-component system, known as the phosphor-relay system comprised of an autophosphorylatable hybrid HK, one or more histidine phosphotransferases (HPT) and a terminal RR was totally absent, because no HPT domains were found in these strains.

### Classification of the histidine kinases

HKs are multi-domain homodimeric proteins consisting of a more variable N-terminal for recognition of diverse signals from different cellular localizations (extracellular, transmembrane or intracellular) and a more conserved C-terminal containing the conserved histidine residue for signal transduction to its corresponding RR protein. The N-terminal regions of most HKs are characterized by the presence of one to several (up to 20) transmembrane helices (although exceptions to this rule could also be found [14]), whereas the C-terminal region harbors characteristic domain profiles such as HATPase_c and His_kinase domains [3,15]. In addition, HK proteins exhibit characteristic sequence motifs (homology boxes), including the H-, N-, D-, F- and G- boxes, as a result of the conservation of the histidine (H), aspargine (N), aspartate (D), phenylalanine (F) and glycine (G) residues at fixed positions. In addition to the H-box, which contains the autophosphorylatable histidine residue functionally essential for a histidine kinase, other homology boxes are also presumed to play crucial roles in different biological processes. For example, the D-box is suggested to be responsible for DNA binding and the G-box is believed to be involved in phosphotransfer reactions. Homology boxes have been used to define various HK families [12]. Thus, to better understand the functions of individual HK proteins of the mutans streptococci strains, two classification approaches were applied in this study to the putative HK proteins. The first approach termed here as homology box analysis was based on the conserved sequence motifs described before by Grebe et al. [12]. This classification scheme sorts HK proteins into 11 different families (HPK1 to HPK11) depending on the sequence motifs of the various homology boxes identified by multi-alignment. Thus, members of the different families can be distinguished by conserved patterns in each of the homology boxes. The second approach termed here as domain architecture analysis was based on the classification scheme proposed by Mascher et al. [14], which considers the conservation of domain architecture of an entire HK, such as transmembrane helices, linker regions, sensing domains and transmitter domains. This classification method is especially helpful in understanding the signal sensing mechanisms of HKs.

### Classification of HKs based on homology box analysis

**Figure 2:**
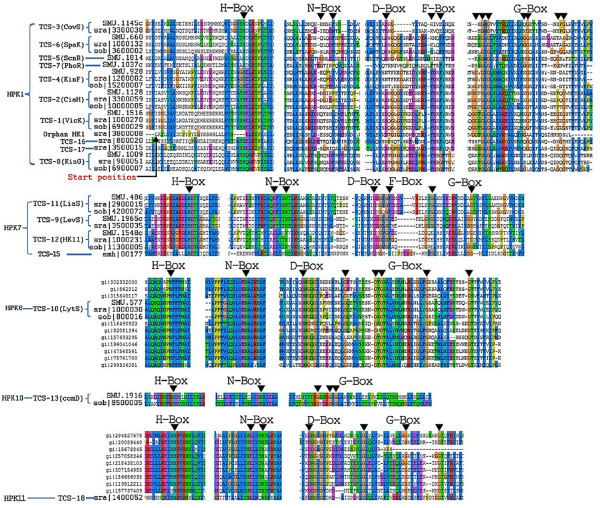
**Multiple-sequence alignments of the regions showing the characteristic homology boxes of the HK proteins**. Because the corresponding HK proteins of the eight *S. mutans *strains in this study were highly similar, *S. mutans *UA159 was used as their representative, wherever possible. The different HK families were obtained as a result of the classification schemes described by Grebe [12].

Five HK families (HPK1, HPK7, HPK8, HPK10, HPK11) were recognized among the 10 mutans streptococci strains. For each of the 10 strains the distributions of their HKs within these families are highlighted in Table 2 and visualized in Figure 2 by multiple-sequence alignments of the HK proteins. It is clear that the amino acid residues surrounding the conserved histidine residue in the H-box are more conserved compared to those flanking the aspargine, aspartate, phenylalanine and glycine residues in the N-, D-, F-, and G-boxes, respectively.

The assignment of HKs of all the 18 TCS clusters to the homology box-based HK families is given in Table 3. HKs of TCS-1 to TCS-8, TCS-16 and TCS-17 and the orphan HK of *S. ratti *DSM20564 (sra|3800008, orphan HK1) were grouped into the HPK1 family, accounting for more than 50% of the total HKs identified. HPK1 family has been known as the most common type among the histidine kinase families, characterized by highly conserved aspartate/glutamate, lysine/arginine and proline residues downstream of the conserved histidine residue in the H-boxes (Figure 2). The orphan HK1 might be a 'dead' gene because of the lack of a sensing domain, which can be inferred from the relatively short N-terminal region prior to the H-box, as highlighted in Figure 2. Moreover, 4 residues including the most important histidine residue in the H-box were also found to be missing in orphan HK1.

The second most abundant HK family was the HPK7 family, which contained more than 20% of all the HKs identified and included HKs of 4 different TCS clusters, namely TCS-9, TCS-11, TCS-12 and TCS-15. As shown in Figure 2, the most prominent feature of this HK family was the conservation of negatively charged aspartate residue downstream of the histidine residue and the positively charged arginine residue upstream of the histidine residue in the H-box.

Two other HK families found were the HPK8 and the HPK10 families. The HPK8 family comprised only HKs of the TCS-10 cluster. Known as LytS in *S. mutans *UA159, HKs of this family were highly conserved across all the 10 strains, as evidenced by the highly conserved sequence patterns of the H- and N-boxes (Figure 2). The identification of HKs belonging to the HPK8 family was made possible by aligning the corresponding HKs of the mutans streptococci strains of this study with their BLASTp homologs. This enabled the unambiguous verification of the conservation of homology boxes characteristic of the HPK8 family. The HPK10 family has been reported to include kinases that regulate competence for genetic transformation in *Streptococcus *spp. [12]. Indeed, HKs of TCS-13 (known as ComD for *S. mutans *UA159) fell within this family. This family was characterized by the presence of a tyrosine located two residues downstream of the conserved histidine residue, the absence of the H-box proline and the lack of the D and F-boxes. HK of TCS-13 (ComD) was absent in *S. ratti *DSM20564, which lacked any orthologs of both ComD and its cognate response regulator ComE.

The last HK family identified is the HPK11 family containing only one histidine kinase uniquely found in *S. ratti *DSM20564. A similar strategy as that used for the classification of HKs of the HPK8 family was performed by using BLASTp homologs to recognize conserved homology boxes. The H-box of this family was quite distinct from the H-boxes of other families in that the conserved histidine was flanked at its left by another histidine residue, in addition to the downstream arginine (R) and asparagine (N) residues characteristic of this family (Figure 2).

The HK of TCS-14 and the unique orphan HK of the strain *S. sobrinus *DSM20742 (sob|13200014, orphan HK2) could not be classified into any of the 11 HK families.

### Classification of HKs based on domain architecture analysis

**Figure 3:**
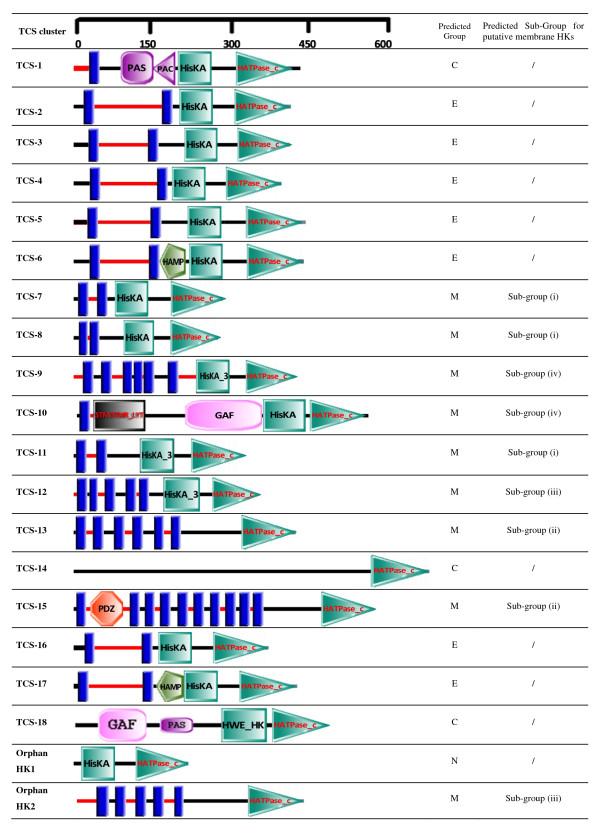
**Domain architectures of histidine kinases representative of each TCS clusters**. The pictorial depiction is based on the predictions carried out using the SMART web interface http://smart.embl-heidelberg.de/ with small manual modifications. The transmembrane helices (TMs), represented by blue bars, and the cellular localization of the linker regions between the helices were predicted using the tool TMHMM [16]. Red and black horizontal lines depict extracytoplasmic and cytoplasmic regions, respectively. The locations of the conserved domains within the protein sequences are represented by the labeled symbols. Domain definitions are according to Pfam http://pfam.sanger.ac.uk: *HAMP*, linker domain presenting in histidine kinase, adenylyl cyclases, methyl binding proteins and phosphatases (PF00672); *HisKA*, dimerisation/phosphoacceptor domain of histidine kinase (PF00512); *HATPase_c*, HK-type ATPase catalytic domain (PF02518); *HisKA_2*, dimerisation/phosphoacceptor domain of a sub-family of histidine kinases (PF07568); *HisKA_3*, dimerisation/phosphoacceptor domain of a sub-family of histidine kinases (PF07730);; *PAS*, signal sensor domain (PF00989); PAC (PF00785), a motif often found at the C-terminal side of the PAS domain and proposed to contribute to the PAS domain fold, *GAF*, cytoplasmic signaling domain (PF01590); *PDZ*, also known as DHR or GLGF (PF00595), a signal sensor domain of diverse functional specificities. All other abbreviations are referred to in Table 2 and Table 3. C, E and M stand for cytoplasmic, extracytoplasmic and membrane sensing, respectively; N denotes no sensing mechanism could be predicted due to either the complete absence or a truncation of the predicted sensory domain.

The homology boxes-based classification of HKs described above focused solely on the conserved sequence motifs in the C-terminal transmitter domains of histidine kinases. Since the biological function of TCS-mediated signal transduction manifests itself in signal input and output rather than in the communication between its two components, it is necessary to characterize the N-terminal sensory regions with respect to the signal sensing mechanism of a particular HK protein. To this end, the classification method proposed by Mascher et al. [14] was applied to group the putative HKs into three different groups: extracytoplasmic-sensing HKs, cytoplasmic-sensing HKs, and membrane-sensing HKs (HKs with sensing mechanisms associated with membrane-spanning helices), as shown in Figure 3.

Extracytoplasmic sensing HKs represent the largest group of membrane-bound sensor kinases. Among all the HKs identified, HKs of TCS-2 to TCS-6 as well as TCS-16 and TCS-17 were recognized as extracytoplasmic sensing HKs by displaying at the N-terminal region an extracytoplasmic putative signal perception domain composed of 110-140 amino acids, which were flanked by (at least) two transmembrane helices (TMs). The cytoplasmic part of the HK proteins harboring the transmitter domain comprised either a HisKA-HATPase_c domain (HKs of TCS-2, TCS-3, TCS-4, TCS-5 and TCS-16) or a HAMP-HisKA-HATPase_c domain (HKs of TCS-6 and TCS-17). HisKA and HATPase_c are domains essential for the functionality of a histidine kinase. HAMP functions as a linker to bridge the transmembrane helix and the transmitter domain [17]. It should be noticed that sequence regions containing about 110 residues were found to exist between the TM region and the HisKA domain of the TCS-3 and TCS-5 HKs. We suspect that these HKs could possibly contain unknown linker domains within this region which cannot be identified due to the currently limited knowledge on HK linker domains.

Cytoplasmic-sensing HKs include either membrane-anchored or soluble proteins with their sensing domains located inside the cytoplasm [14]. Only HKs of three TCS clusters, namely the membrane anchored HK of TCS-1 and the soluble HKs of TCS-14 and TCS-18, were identified as HKs with putative cytoplasmic sensing functions. A more detailed look at the HKs of TCS-14 and TCS-18 showed that they were predicted to be completely intracellular proteins. The HK of TCS-1, known as VicK in *S. mutans *UA159, is typified by the presence of only one membrane-anchoring transmembrane helix and a very short extracytoplasmic linker containing 30 amino acids in the N-terminal region. In addition, both HKs of TCS-1 and TCS-18 possess a PAS domain. PAS domains are known to play important roles as sensory modules for sensing oxygen tension, cellular redox state, or light intensity [18]. PAS domain-containing proteins have been reported to be involved in oxidative stress response [19], competence [20], virulence [21] and biofilm formation [22] in many organisms. Most PAS domain-containing proteins are intracellularly located with dual functions of monitoring both the external and internal environments by perceiving alterations in the electron transport system caused by intracellular or extracellular changes in redox potential [23]. It has been reported that PAS domains are often associated with PAC domains and they are directly linked and together form the conserved 3D PAS fold [24], as also exemplified by the HK of TCS-1 in this study. In fact, in agreement with the function of the PAS-PAC domains, the HK of TCS-1, VicK, has been reported to play a role in oxidative stress response [25]. On the other hand, the HK of TCS-18 did not contain a PAC motif but a GAF domain, which has been found to be present in combination with multiple domains in many phytochromes and cGMP-specific phosphoesterases [26]. A large variety of bacterial proteins such as those involved in signal transduction, cell autolysis, chemotaxis, glucose metabolism, nitrogen fixation, and gene regulation, as well as phototransducing proteins in plant and archea were found to contain a GAF domain [27].

The highly diverse group of HKs with sensing mechanisms linked to transmembrane regions (membrane-sensing HKs) is marked by the presence of 2 to 20 transmembrane helices implicated in signal perception. Such HKs lack a recognizable extracellular input domain. Signal sensing is either membrane associated or occurs directly within the membrane interface [14]. Although it has been suggested that this group represents the smallest group of membrane-bound sensor histidine kinases when compared to other HK families in the SMART database [14], HKs of TCS-7 to TCS-13 and TCS-15 in this study were all found to belong to this group, indicating that a relatively high percentage of HKs of the mutans streptococci strains are involved in sensing signals directly associated with the membrane. Such signals can be changes in the mechanical properties of the cell envelope, signals derived from membrane-bound enzymes or other membrane-integral components, ions, electrochemical gradients, transport processes, or the presence of compounds that affect cell envelope integrity [14]. Most quorum sensing HKs from gram-positive bacteria fall into this category, like ComD, the HK of TCS-13. Putative alleles/orthologs of ComD were found in most of the mutans streptococci strains of this study, characterized by 5 to 7 transmembrane helices in the N-terminal region succeeded by a transmitter region containing only the HATPase_c domain. In *S. sobrinus *DSM20742 the unique orphan HK2 (sob|13200014) was also assigned to this group. Orphan HK2 shared a sequence similarity of about 37% with the HK (sob|8500005) of TCS-13, which is the predicted ortholog of ComD in *S. sobrinus *DSM20742.

For the highly diverse membrane-sensing HK family, six subfamilies have been additionally defined based on features such as the number of TM helices, the type and sequential arrangement of sensory domains, the length of the inter-helix linker regions and the presence or absence of extracytoplasmic linker regions [14]. By applying this grouping scheme, the membrane-sensing HKs of TCS-7, TCS-8 and TCS-11 were grouped into subgroup (i). This subgroup is comprised of small sensor kinases containing 2 TM helices and lacking significant extracytoplasmic regions. HKs of this subgroup are involved in sensing cell envelope stress or mediating ABC transporter-coupled detoxification processes in gram-positive bacteria [28,29]. HKs of TCS-13 and TCS-15 were assigned to subgroup (ii), which is primarily comprised of quorum-sensing HKs of gram-positive bacteria possessing 6 to 10 TM helices. HKs of TCS-12 and the orphan HK2 were grouped into subgroup (iii) represented by DesK-like thermo sensors with 4 or 5 TM helices that sense membrane fluidity. HKs of TCS-9 and TCS-10 were assigned to subgroup (iv) with a conserved but yet functionally unknown input domains containing 6 to 8 TM helices.

### Classification of the response regulators

A RR protein, most commonly a transcription factor [3], comprises an N-terminal regulatory domain (receiver domain) and a C-terminal effector domain (output domain). The phosphorylated aspartate residue falls within the receiver domain whose structure is well conserved as represented by the Response_reg domain among almost all the known RRs [30]. Phosphorylation of the aspartate residue activates the DNA binding properties of the output domain, ultimately resulting in the direct or indirect activation or repression of a set of genes. The output domains are less conserved and differ according to the nature of the signal outputs. Hence, a classification of RRs based on the domain architecture of the C-terminal effector domain should provide insight into the specific biological function of the TCSs [31]. Despite the great variety of output domains and domain combinations, about 60% of all known response regulators belong to the OmpR, NarL or NtrC families, and all of which contain a helix-turn-helix (HTH) DNA-binding output domain. RRs with the DNA-binding output domains LytTR, AraC, Spo0A, Fis, YcbB, RpoE, and MerR account for an additional 6% [30]. The rest of the RRs are characterized by the presence of a stand-alone REC domain (14%) or combinations (20%) comprising a variety of enzymatic domains, RNA-binding domains, protein or ligand-binding domains, or other newly described domains of unknown functions. The diversity of the domain architectures and the abundance of domain combinations of the output domain allow bacterial cells to regulate a variety of biological processes such as transcription, enzyme activity and protein-protein interaction in response to a number of environmental signals and stimuli [31].

The majority of the putative RRs identified in this study were classified into the following 4 families: NarL, LytTR, AmiR and OmpR, with RRs of the OmpR family constituting the largest group. For each of the 10 compared strains the distributions of their RRs within these families are highlighted in Table 2. The assignment of RRs of the 18 TCS clusters and the orphan RRs to the corresponding RR families is given in Table 3.

While most RRs contain a DNA binding domain, there were two exceptions: the putative RR of TCS-14 lacked any currently known output domains and therefore could not be assigned to any of the known RR families. The RR (sra|1400053) of TCS-18, a unique TCS cluster of *S. ratti *DSM20564, contained a RNA-binding output domain and was found to be an AmiR-like response regulator. The AmiR family is a relatively newly defined response regulator family possessing RNA-binding activity. In contrast to the commonly known phosphorylation mechanism in DNA-binding RRs, AmiR is normally controlled by ligand-regulated activation, in keeping with the substitution of the conserved aspartate phosphoryl acceptor residue by serine, as well as the replacements of additional amino acid residues required for phosphoryl transfer [32]. However, unlike the classical AmiR-like proteins, residues involved in phosphoryl transfer are conserved in the RR of TCS-18. In addition, we have identified its cognate HK (sra|1400052). Therefore, we believe that RR of TCS-18 is most likely activated by its cognate HK through phosphorylation.

RRs of the OmpR family constituted the largest group (Table 2). Proteins of the OmpR family have been reported to mediate a wide range of biological functions related to, for example, osmolarity, phosphate assimilation, antibiotic resistance, virulence and toxicity [33]. NarL proteins differ from those of the OmpR family due to the presence of the LuxR_C-like DNA-binding HTH domain. Members of the NarL family have been documented to control expression of genes related to nitrogen fixation, sugar phosphate transport, nitrate and nitrite metabolism, quorum sensing, and osmotic stress etc. [34]. Proteins of the LytTR family are characterized by having a non-HTH DNA binding domain [35]. They account for about 3% of all the prokaryotic RRs [31]. Despite being found in prokaryotic genomes in small numbers when compared to other RR families, the LytTR type RR proteins have been reported to modulate the expression of many genes coding for virulence factors, fimbriae, cell wall components, bacteriocins, extracellular polysaccharides etc. [36]. Also of interest is the fact that RRs belonging to NtrC, AraC, Spo0A, Fis, YcbB, RpoE and MerR families, which often appear in other bacterial genomes, are totally absent in the mutans streptococci strains compared in this study.

### Co-occurrence of certain HK and RR families

The homology box-based classification of HK proteins has been shown to be able to reveal the co-occurrence relationships between the various kinase families and regulator families [12]. In other words, specific families of HK were preferentially associated with specific families of RR proteins. In this study, certain pairing combinations between the HK families and the RR families were also observed in the mutans streptococci strains as shown in Table 3. For instance, all HKs of the HPK1 family are paired with corresponding RRs of the OmpR family, and all HKs of the HPK7 family constitute TCS clusters with RRs of the NarL family. HKs of both the HPK8 and HPK10 kinase families prefer RRs of the LytTR family as their corresponding cognate response regulators. We reckon that such combinations may play a significant role in the phenomenon of cross-talking between unpaired HKs and RRs, e.g. a HK of the HPK1 family is highly likely to cross-phosphorylate an unpaired RR of the OmpR family. Cross-talking mechanisms have been reported to exist in many bacterial species [37] and have also been speculated to appear in *Streptococcus mutans *strains [38]. Such a strategy could be advantageous for mutans streptococci strains to maximally utilize their relatively small complement of TCSs for processing diverse signals so as to ensure a flexibly coordinated response to the constantly changing environments. For example, while a viable knock-out mutant of the *vicK *gene coding for the HK of TCS-1 could be obtained, the cognate response regulator VicR has been found to be essential for the survival of *S. mutans *UA159 [39]. It has also been reported that no viable *vicR *knockout mutants could be obtained for *Streptococcus pneumoniae*, but knockout of the corresponding histidine kinase gene *vicK *is possible, because *vicR *can be phosphorylated *in vivo *by *vanS*, a gene coding for a TCS histidine kinase from *Enterococcus faecium*, which helps to suppress some effects of a *vicK *kinase disruption in *S. pneumoniae *[40]. According to homology box analysis the histidine kinase VanS of *Enterococcus faecium *belongs to HPK1 family like VicK. Thus, we speculate that such cross-phosphorylation of VicR by a non-cognate HK most likely also holds true in *S. mutans *UA159, and probably also for other mutans streptococci strains. Clearly, the systematic classification of HKs and RRs carried out in this study reveals potential cross-talking candidates that will be helpful in the future studies of two-component systems.

### Putative biological functions of the identified TCS proteins

In the following sections, possible biological functions and significance of the TCS genes/proteins, which were common or specific to the 10 mutans streptococci strains as presented in Table 3, are discussed. To this end and for the purpose of a better overview, we have briefly summarized available knowledge on the biological functions of the TCS genes/proteins of the best studied *S. mutans *strain UA159. In case no knowledge is available about the functions of some TCSs, information about putative orthologs of the corresponding TCS genes/proteins in organisms other than *S. mutans *UA159 are provided (Table 4).

**Table 4:** A brief summary of known/putative functions of the TCSs identified in *S. mutans *UA159

TCS Cluster	TCS protein	GenBank Locus Tag	Functions	References
TCS-1	HK-VicK RR-VicR	SMU.1516 SMU.1517	Biofilm development, competence development, oxidative stress tolerance, acid tolerance, autolysin production, glucan metabolism, fructan metabolism.	[7,8,10,25,41-44]

TCS-2	HK-CiaH RR-CiaR	SMU.1128 SMU.1129	Sucrose-dependent biofilm formation, competence development, multiple stress response, bacteriocin production	[13,45,46]

TCS-3	HK-CovS RR-CovR	SMU.1145 SMU.1146	Acid tolerance, hydrogen peroxide resistance, murine macrophage killing.	[13,47]

TCS-4	HK-KinF RR-LlrF	SMU.928 SMU.927	Acid tolerance, pp(G)pp metabolism, control of alarmone synthesis.	[48,49]

TCS-5	HK- ScnK RR- ScnR	SMU.1814 SMU.1815	Bacteriocin production*.	[50]*

TCS-6	HK- SpaK RR- SpaR	SMU.660 SMU.659	Bacteriocin production*, self-protection against anti-microbial peptides*.	[51-53]*

TCS-7	HK- PhoR RR-YcbL	SMU.1037 SMU.1038	Unknown.	

TCS-8	HK-KinG RR-LlrG	SMU.1009 SMU.1008	Bacteriocin resistance, substrate transport in cell envelope stress.	[54,55]

TCS-9	HK-LevS RR-LevR	SMU.1965 SMU.1964	Biofilm formation, acid tolerance, fructan metabolism.	[56,57]

TCS-10	HK-LytS RR-LytT	SMU.577 SMU.576	Biofilm formation, oxidative stress tolerance, autolysis, fructan metabolism, cell wall metabolism*.	[58]*, [49,59]

TCS-11	HK-LiaS RR-LiaR	SMU.486 SMU.487	Biofilm formation, acid tolerance, cell envelope stress response, bacteriocin production, bacteriocin resistance, sucrose-dependent adherence.	[38,60-63]

TCS-12	HK-HK11 RR-RR11	SMU.1548 SMU.1547	Unknown.	

TCS-13	HK-ComD RR-ComE	SMU.1916 SMU.1917	Biofilm formation, quorum sensing, competence development, bacteriocin production.	[9,45,64-66]

TCS-14	HK RR	SMU.45 SMU.46	Unknown.	

Orhpan RR1	RR-GcrR	SMU.1924	Sucrose-dependent adherence, glucan metabolism, pH homeostasis, competence development.	[67-73]

In case no knowledge is available on the functions of some TCSs in UA159, reports on homologous TCSs in other microorganisms, preferably *Streptococcus *species (highlighted by the astral sign*) are provided.

### TCS proteins common to all the 10 mutans streptococci strains

Proteins of the TCS clusters 1, 2, 4, 6, 8, 10, 11, 12 and the orphan RR1 are common to all the 10 mutans streptococci strains compared here, indicating probably the functional importance of these TCS clusters for the adaptation and survival of these mutans streptococci (Table 4). For instance, Orphan RR1 is highly conserved across the 10 mutans streptococci strains. In *S. mutans *UA159, this orphan RR is encoded by *gcrR *(SMU.1924c) and has been found to play a vital role in sucrose-dependent adherence and cariogenesis [73]. Therefore, it is conceivable that conservation of this gene across the mutans streptococci strains is essential for their primary pathogenicity.

### TCS proteins uniquely present/absent in one or several strains

**Figure 4:**
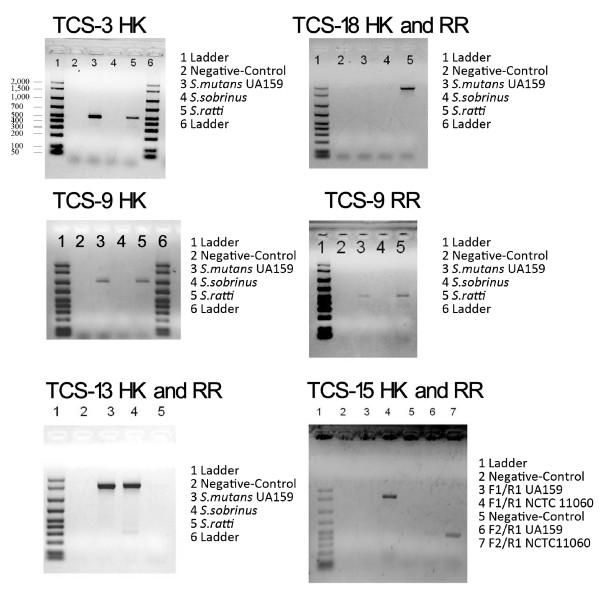
**PCR verifications of the unique presence or absence of TCS genes**. The unique absence of TCS-3 and TCS-9 in *S. sorbrinus *DSM20742 are demonstrated by showing the absences of the HK of its TCS-3 and the HK and RR of its TCS-9, in contrast to the presence of the corresponding genes in other strains, as exemplified in *S. mutans *UA159 and *S. ratti *DSM20564. The absence of TCS-13 and the presence of TCS-18 in *S. ratti *DSM20564 are presented in contrast to *S. mutans *UA159 and *S. sorbrinus *DSM20742. The unique presence of TCS-15 in *S. mutans *NCTC11060 was verified using two forward primers. The sequences of the primers used for PCR are provided in the **Materials and Methods **section.

The TCS-3 (CovSR) cluster was predicted to be absent in *S. sobrinus *DSM20742. CovSR is involved in the acid tolerance response of *S. mutans *UA 159 [13], and has also been reported to be involved in counteracting oxidative stress and reducing susceptibility to phagocytic killing [47]. TCS-9 (LevRS), which affects the acid tolerance response as well [13], was also absent in *S. sobrinus *DSM20742. The absence of the *covS *and *levS *genes was experimentally supported by the PCR results shown in Figure 4. In *S. mutans *UA159, the *levRS *gene cluster is flanked *by levQ *and *levT*, which code for two carbohydrate-binding proteins. These four genes together constitute a four-component signal transduction system *levQRST *controlling the transcription of the fructan hydrolase gene (*fruA*) and a four-gene cluster *levDEFG*, which encode a fructose/mannose sugar:phosphotransferase system located immediately downstream of *levQRST *[56]. *S. sobrinus *was also found to lack the *levQ*, *levT *and *levDEFG *genes. Taking together, these findings indicate dramatic differences in the regulation of fructan catabolism and the acid tolerance response of *S. sobrinus *DSM20742 in comparison to the *S. mutans *strains.

It should be pointed out that the TCS nomenclature is unfortunately inconsistent among the published articles on TCSs of *S. mutans *strains. Many publications [67-72] on CovR actually address the orphan response regulator RR1 which is known as GcrR (SMU.1924) in *S. mutans *UA159 [73]. On the other hand, CovSR of *S. mutans *has also been confusingly named as ScnKR [13,47]. In our study, the nomenclature of TCS genes/proteins was based primarily on the Oralgen Pathogen Sequence Database http://www.oralgen.lanl.gov. In cases where several names were given for a gene in Oralgen, gene names used by Biswas et al. [6] were preferably used.

TCS-5 (ScnKR) could be neither found in the two *S. mutans *strains ATCC25175 and NCTC11060 nor in *S. ratti *DSM20564 and *S. sobrinus *DSM20742. The asymmetric distribution of TCS-5 was also observed in a previous study, in which TCS-5 was found to be present only in two of the 10 *S. mutans *strains compared [6]. In *S. mutans *UA159, an insertion mutant of *scnK *gene displayed no significant difference to the wild type with respect to growth under various stress conditions [6]. In *Streptococcus pyogenes*, ScnKR was found to be essential for the production of a bacteriocin (SAFF22) [50]. By a closer look at the genes in the neighborhood of ScnKR in the 10 strains studied, we found that *S. mutans *KK23 and *S. mutans *NN2025 carried two and three genes, respectively, which encode putative bacteriocin precursor peptides sharing more than 60% similarity with SAFF22. In addition, a putative bacteriocin biosynthesis protein coding gene was also found downstream of the *scnKR *operon in the two *S. mutans *strains. We therefore infer that TCS-5 (ScnKR) might be involved in the regulation of mutacin production at least in *S. mutans *KK23 and *S. mutans *NN2025.

TCS-7 (PhoR/YcbL) was only shared by the eight *S. mutans *strains. In *S. mutans *UA159, an insertion mutant of the gene encoding PhoR displayed no significant difference to the wild type with respect to growth under various stress conditions [6]. The clear function of TCS-7 is still unknown.

As mentioned before in the identification and classification of TCS proteins, TCS-13 (ComDE) was absent in *S. ratti *DSM20564. This finding was also supported by the PCR experiment (Figure 4). In *S. mutans*, ComDE is the most extensively studied two-component signaling system involved in quorum sensing and competence development. Mediated by the so-called competence stimulating peptide (CSP), it is involved in multiple stress responses and has been implicated in competence development, bacteriocin production, virulence, biofilm formation, and cariogenicity [9,45,64-66]. Further analysis showed that the ortholog of the *comC *gene, whose product is the precursor of the signal peptide CSP sensed by ComDE in *S. mutans*, was absent in *S. ratti*. Putative orthologs of *comD *and *comE *were found in *S. sobrinus *DSM20742. However, with a similarity of merely 37% and 43%, respectively, to the *comD *and *comE *of *S. mutans *UA159 (Table 3), it's highly possible that the actual function of TCS-13 in *S. sobrinus *DSM20742 might be quite different from that known for *S. mutans*. It is also worthy to mention that no *comC*-like gene was found in *S. sobrinus *DSM20742.

Putative alleles of the HK and RR proteins of TCS-14 are present in five of the *S. mutans *strains, namely 5 DC8, KK23, KK21, UA159 and NN2025 (Table 3). This cluster was first identified in *S. mutans *UA159 by Biswas et al. and the corresponding HK and RR are encoded by SMU.45 and SMU.46, respectively [6]. They also found that TCS-14 was present only in two of the 13 *S. mutans *strains compared in their study. HKs of this TCS cluster contain only one recognizable HATPase_c domain (Figure 3). In addition, no known output domain was identified in the cognate RRs. Thus, neither HKs nor RRs of TCS-14 could be classified into any known HK and RR families. In addition, as depicted by the multi-sequence alignment of the putative TCS-14 HK alleles in Additional file 1, the open reading frame predictions carried out in our study revealed that while SMU.45 and its upstream gene SMU.44 clearly constitute two separate genes in *S. mutans *UA159, they are merged to constitute the parts of a single gene coding for the HKs in the *S. mutans *strains 5 DC8, KK23 and NN2025. The corresponding HK of KK21 was also split into two proteins by the lacking of a single glutamine (Q) residue.

TCS-15 was found exclusively in the genome of the serotype f blood isolate *S. mutans *NCTC11060 (Table 3). Genes of a TCS located on the genomic island TnSmu2 of *S. mutans *UA140, as recently reported by the research group Qi [74], could be possible alleles of TCS-15 genes. This was based on the fact that the predicted HKs and RRs of both TCS 15 in our study and the TCS found by Qi's group have the HK (YP_002747386.1) and RR (YP_002124238.1) of *Streptococcus equi*, respectively, as the best matched homologs. The HK (smh|00177) of TCS-15 is also the only histidine kinase found in this study that contains a PDZ domain (Figure 3). PDZ domain was first reported to be present in animals. In 1997, Ponting et al. claimed that PDZ domains exist also in diverse signaling proteins of bacteria, yeasts and plants. Experimental evidence was first provided by Liao et al. through the determination of the first crystal structure of a bacterial PDZ domain [75]. The most remarkable feature of PDZ domains is their ability to specifically recognize and bind to short C-terminal peptide motifs. This allows them especially to bind membrane proteins such as ion channels, which have very small free C-termini. To exclude the possibility of contamination with e. g. human DNA during the process of genome sequencing, the existence of the gene coding for this unusual PDZ domain-containing HK protein in the DNA of the NCTC11060 strain was experimentally verified by using two different forward primers in the PCR experiment (Figure 4).

The PDZ domain of smh|00177 is flanked by one transmembrane helix (TM) at its N-terminal side and 9 TMs at its C-terminal side, which is characteristic of a ComP-like HK. ComP-like HKs are a group of sensors of another peptide-dependent quorum sensing system related to cell density-responsive regulation other than ComDE in gram-positive bacteria. In *B. subtilis*, ComP is the sensor histidine kinase of the four-component *comQXPA *quorum sensing system, where ComA stands for the corresponding response regulator, ComX is the pheromone precursor and ComQ the protein required for the proteolytic cleavage and modification of the pheromone precursor molecule [76]. The most similar homolog of smh|00177 found from a BLASTp search in the NCBI database http://blast.ncbi.nlm.nih.gov/ with a sequence identity of around 65% is the histidine kinase (YP_003353659.1) of a TCS from *Lactococcus lactis *subsp. *lactis *KF147. Furthermore, we found that the cognate response regulator of TCS-15 showed a sequence identity of around 78% with the corresponding response regulator (YP_003353660.1) from the same *L. lactis *subsp. *lactis *strain. In our study, the RR (smh|00177) of TCS-15 was termed as CmpR (Table 3). It should be mentioned that neither *L. lactis *subsp. *lactis *KF147 nor *S. mutans *NCTC11060 possesses homologs of the *B. subtilis comX *and *comQ*. Thus, the signal peptide sensed by ComP/CmpR in *S. mutans *NCTC11060 remains unknown.

TCS-16, 17 and 18 are uniquely present in *S. ratti *DSM20564 (Table 3). According to BLASTp searches against the NCBI database, the top matches to the TCS-16 HK protein (sra|800020) or the RR protein (sra|800019) are all from *Streptococcus *species such as *Streptococcus infantarius *and *Streptococcus agalactiae*. In addition, the HK and RR homologs in the different *Streptococcus *species are all encoded by two adjacent genes and annotated in some *S. agalactiae *strains as sensor histidine kinase DltS and DNA-binding response regulator DltR. The DltSR has been reported to be involved in the regulation of D-alanyl-lipoteichoic acid biosynthesis in *S. agalactiae *[77]. Lipoteichoic acid (LTA) is a major cell wall constituent of Gram-positive bacteria which is phosphoglycerol substituted with a D-Ala ester or a glycosyl residue and anchored in the membrane by its glycolipid moiety. D-alanylation of lipoteichoic acid has been proven to contribute to the virulence of *Streptococcus suis *[78], as well as to the biofilm formation and resistance to antimicrobial peptides in enterococci [79]. Thus, the TCS-16 cluster might also be an important virulence factor in *S. ratti*.

The TCS-17 was composed of a HAMP-containing HK and an OmpR-type RR. The best homologs of the HK protein (sra|3500015) and the RR protein (sra|3500014) are from *S. agalactiae *strains. But the functions of these homologous proteins remain unknown.

TCS-18 is the only cluster that comprises a HPK 11 family HK and an AmiR family RR, which possesses a novel RNA-binding type output domain. The top ten best hits of the BLASTp search in the NCBI database showed that the closest homologs for the HK and RR of TCS-18 are all from *Listeria *species. Since the HK of TCS-18 possesses a PAS domain that is commonly involved in sensing intracellular signals such as redox potential, similar to the sensing mechanism described for the HK (VicK) of TCS-1, the function of TCS-18 might also be related to the sensing and response to signal(s) originated in the cytoplasm. The unique presence of TCS-18 in *S. ratti *DSM20564 was also confirmed by the PCR experiment (Figure 4).

## Conclusions

In the present study we conducted a genome-wide identification, classification, and ortholog analysis of the TCS proteins in eight newly sequenced mutans streptococci strains in comparison with two previously sequenced *S. mutans *strains, UA159 and NN2025. Totally, 18 TCS clusters comprising HK-RR pairs were identified, with 8 of them shared by all the 10 strains compared, 6 being absent in one or more strains, 1 unique to *S. mutans *NCTC11060 and 3 exclusive to *S. ratti *DSM20564. *S. mutans *strains share to a large extend the same TCS repertoires. One remarkable exception is the unique presence of the putative quorum-sensing TCS-15 (*ComP/CmpR*) in the only serotype f blood isolate, *S. mutans *NCTC11060. *S. sobrinus *DSM20742 shows differences to the *S. mutans *strains in the signal perceptions possibly related to fructan catabolism and acid tolerance response. With its 3 unique TCS clusters, *S. ratti *DSM20564 seems even more distinct to the *S. mutans *strains. One of the unique TCS clusters is homologous to the DltSR TCS in *Streptococcus agalactiae *strains that is probably involved in the regulation of the biosynthesis of D-alanyl-lipoteichoic acid, an important cell wall component. Another one has an AmiR-like RR protein, which is the only RR found in this study to contain a RNA-binding output domain instead of a DNA-binding output domain.

In addition to highlighting putative functions of the individual histidine kinases and response regulators, our detailed classification of the TCS proteins also confirms that specific HK families are preferentially associated with specific RR families. Such associations might play significant roles in the phenomenon of cross-talking between unpaired HKs and RRs.

The asymmetric distribution of TCS among the mutans streptococci strains compared in this study implies that *S. mutans*, *S. sobrinus *and *S. ratti *might employ different sensing and response mechanisms for their survival in the fast changing oral environment, mostly in a symbiotic lifestyle. We believe that the results from this genomic level study will be certainly helpful for the design of physiological studies and mutation analysis towards the verification of some hypotheses proposed in this study, which in turn will lead to a better understanding of signal transductions involved in the pathogenicities of mutans streptococci.

## Methods

### Mutans streptococci strains sequenced

We have recently sequenced eight mutans streptococci strains as given in Table 1 using the Solexa sequencing platform at the Helmholtz Center for Infection Research in Braunschweig, Germany. The "high-quality draft" genome sequences of these mutans streptococci strains were assembled by a combined use of the sequence assembly tools SOAPdenovo [80], Maq [81] and Phrap [82]. Corresponding protein sequences were predicted using Glimmer3.02, a gene finding tool based on interpolated Markov models [83]. Detailed genome sequence information is available at http://134.28.64.65/www/index.php/genome.

### Identification of the histidine kinases and response regulators of putative two-component signal transduction systems

The identification of histidine kinases (HKs) and response regulators (RRs) of putative two-component systems (TCSs) of the eight mutans streptococci strains was carried out based on computational domain analysis of the predicted protein sequences. Two previously sequenced *S. mutans *strains, the *S. mutans *UA159 and *S. mutans *NN2025, were used as reference strains for comparison. To this end, the same identification procedure was carried out on the genomes of *S. mutans *NN2025 and UA159 to ensure that the same search criteria were applied for all the strains included in this study so that a reasonable comparison can be achieved. The genome sequences of the two reference strains were obtained from the genome database at the National Center for Biotechnology Information http://www.ncbi.nlm.nih.gov/sites/genome. Approaches for identifying HKs and RRs were similar to those described previously [84] with slight modifications. Briefly, putative HK and RR proteins were identified by Hidden Markov Model (HMM) searches using the related HMM profiles available in the Pfam database http://pfam.sanger.ac.uk/ as templates [15]. The sequence homology search software HMMER3 http://hmmer.org/[85] was used for scanning the predicted protein sequences with the HMM profiles. All the HK related HMM profiles with the accession numbers PF00512, PF07568, PF07730, PF07536, PF06580, PF01627, PF02895, PF05384, PF10090 were used for identifying putative HKs. The HMM profile PF00072 which targets the receiver (REC) domain of RR proteins was used to recognize putative RRs. For the identification of HKs, the homology search was performed without setting E-value/score cutoffs to avoid missing any putative HKs with low scores. However, all the identified putative HKs were manually validated by judging whether at least one of the following two criteria was satisfied: (a) the presence of a cognate putative RR in the same operon as the putative HK in question; (b) the presence of both the HisKA-like and HATPase_c domains so that any HATPase_c domain possessing non-HK proteins could be excluded. For the identification of putative RRs, the E-value cut-off was set at 1e-6. Paired HK and RR present in the same operon comprise a TCS cluster. Hybrid HKs, if any, could be determined by the presence of a complete HK transmitter domain and a REC domain in a single protein. HKs and RRs are defined as orphan HKs or RRs, if no corresponding cognate RRs or HKs can be found in the same operon. The operon information used in this study was predicted by Pathway Tools [86].

### Identification of common and unique TCS proteins

TCS proteins that are common or unique among the mutans streptococci strains were identified through ortholog analysis. The ortholog groups were constructed by using the OrthoMCL program [87]. OrthoMCL was first developed by focusing on eukaryotic genomes and now has been successfully applied to prokaryotic genomes as well. BLASTp [88] was employed to construct the homologous protein matrices which were used in the ortholog group identification process by OrthoMCL with an E-value cutoff of 1e-5.

### Classification of the TCS proteins

Functional domains of the TCS proteins, which were needed for the classification of both HKs and RRs were obtained by subjecting the predicted proteome of each strain to a search against the protein sequence analysis and classification database InterPro using the protein signature recognition software InterProScan [89]. In addition, for the classification of HK proteins, transmembrane helices were predicted by using the hidden Markov model based program TMHMM2 [16].

The HK protein classifications were carried out by two different approaches: a) homology box based analysis through the recognition and alignment of characteristic sequence motifs (homology boxes) of different HK families, as described previously by Grebe et al. [12]; b) domain architecture based analysis of the transmembrane regions and domain organization by a method described previously by Mascher et al. [14]. As the SMART database http://smart.embl-heidelberg.de provided a user-friendly web interface and excellent illustration features, the visualization of HK transmembrane helices, conserved domains and their organization was realized by using the SMART web interface with small manual modifications for the pictorial depictions.

### Co-evolution analysis of paired HKs and RRs

All the putative HKs and RRs identified in the mutans streptococci strains compared in this study were used for establishing the phylogenetic relationships between the TCS proteins. Using the ClustalX program multiple alignments were performed with the following parameter settings: residue-specific penalties: on, hydrophilic penalties: on, gap separation distance: 4 and the phylogenetic relationships with a bootstrap value set at 1000 were constructed [90]. The results were imported into the visualization software Treeview [91] for the visualization of the phylogenetic trees of HKs and RRs, respectively.

### PCR verification

To verify the unique presence of TCS-15 in *S. mutans *NCTC11060 and to exclude the possibility of contamination with e. g. human DNA during the process of genome sequencing, PCR amplification with original DNA from this strain (and from strain *S. mutans *UA159 as negative control) using two different forward primers was performed. The primers used were: 5'-TTGCTTGCTGTTGTTGTG-3' (forward primer), 5'- GGCTACCATTTAGTAGAAAAGAGG -3' (alternative forward primer), 5'-TGTTACCATCTTCGGAAGG-3' (reverse primer), which were designed by using Primer Premier 6 http://www.premierbiosoft.com/primerdesign/index.html and Vector NTI 9.0 (InforMax) respectively. Conditions for this conventional PCR were: 94°C, 2 min; followed by 32 cycles of 94°C for 30 s; annealing temperature 49°C for 30 s; and 72°C for 90 s; final extension at 72°C for 5 min; length of amplicons: 1624 bp and 504 bp, respectively.

To verify the unique presence of TCS-18 in *S. ratti*, the unique absence of TCS-13 in *S. ratti*, and the unique absence of TCS-9 as well as TCS-3 in *S. sobrinus*, PCR amplification using original DNA from strains *S. mutans *UA159, S. *ratti *DSM20564, and *S. sobrinus *DSM20742 was performed. The primers used, the annealing temperatures and the lengths of amplicons were as follows (all other parameters were kept as mentioned above): TCS-18 F 5' CACTGTTCCTCCTGTATCC 3', TCS-18R 5' ATGCTGGCTATGATGTTGT 3' (T_a _= 50°C, length: 1899 bp covering HK and RR); TCS-13 F 5' RAKTTYATGCCYCTMACYTTYCAG 3', TCS-13R 5' GATTCRWWRGCMGCCTC 3' (T_a _= 49°C, length: ~1600 bp covering HK and RR); TCS-9 HK-F 5' ATACAGTCAATATGCYAAGC 3', TCS-9HK-R 5' GRATAACACGGAAAA 3' (T_a _= 45 C, length: 1055 bp); TCS-9 RR-F 5' TGCTGARGACCAAGA 3', TCS-9RR-R 5' TTAGCTGCAATTTCTT 3' (T_a _= 50°C, length: 522 bp); TCS-3 HK-F 5' CAYGAYYTIMGIAAYCC 3', TCS-3 HK-R 5' GTDATIACIGTICCC 3' (T_a _= 40°C, length: 505 bp).

## Abbreviations

TCS: Two component system; HK: Sensing histidine kinase; RR: Response regulator; HTH: Helix-turn-helix; HMM: Hidden Markov Model; HPT: Histidine phosphotransferases; TM: Transmembrane helix; HGT: Horizontal gene transfer; PCR: Polymerase Chain Reaction.

## Competing interests

The authors declare that they have no competing interests.

## Authors' contributions

LS carried out the major bioinformatics analysis and participated in drafting the manuscript. PS was involved in the bioinformatic analysis and contributed significantly to drafting the manuscript. WW participated in the conception and coordination of the study and contributed significantly to drafting the manuscript. GC and AB performed the PCR verification experiments. JS was involved in genome sequencing and annotation. GC and IWD contributed to the microbial aspects and valuable discussions. AZE conceived of and supervised the study and revised the manuscript. All authors read and approved the final manuscript.

## Supplementary Material

Additional file 1 Multiple sequence alignment of the allelic HK proteins belonging to the TCS-14 cluster. smd|00044 and smd|00045 from *S. mutans *KK21; sme|00049 from *S. mutans *KK23; smc|00046 from *S. mutans *5DC8; SMU.44 and SMU.45 from *S. mutans *UA159; GI|290579565 from *S. mutans *NN2025. The splitting of the alleles into two separate proteins in the strains *S. mutans *KK21 and *S. mutans *UA159 can be observed. Click here for file
