# Supplementary figures and images for: A genome-wide study of two-component signal transduction systems in eight newly sequenced mutans streptococci strains
Source: BMC Genomics. 2012 Apr 4;13:128. doi: 10.1186/1471-2164-13-128 (PMC3353171; doi:10.1186/1471-2164-13-128)

###
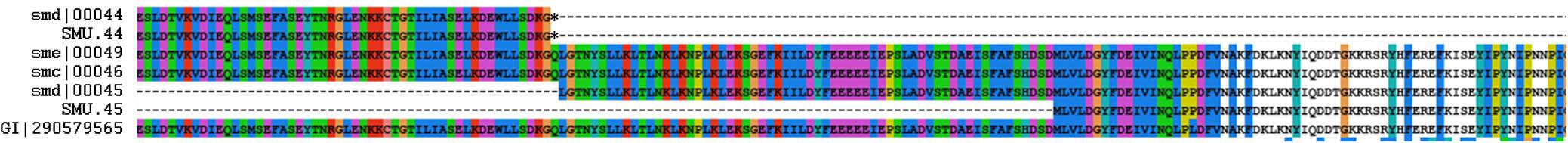

Supplement: Additional file 1 — Multiple sequence alignment of the allelic HK proteins belonging to the TCS-14 cluster. smd|00044 and smd|00045 from S. mutans KK21; sme|00049 from S. mutans KK23; smc|00046 from S. mutans 5DC8; SMU.44 and SMU.45 from S. mutans UA159; GI|290579565 from S. mutans NN2025. The splitting of the alleles into two separate proteins in the strains S. mutans KK21 and S. mutans UA159 can be observed. [file 1471-2164-13-128-S1.DOC]
